# Magnetic Interaction of Multifunctional Core–Shell Nanoparticles for Highly Effective Theranostics

**DOI:** 10.1002/adma.201802444

**Published:** 2018-10-11

**Authors:** Ming-Da Yang, Chien-Hsin Ho, Sergiu Ruta, Roy Chantrell, Kathryn Krycka, Ondrej Hovorka, Fu-Rong Chen, Ping-Shan Lai, Chih-Huang Lai

**Affiliations:** Department of Materials Science and Engineering, National Tsing Hua University, No. 101, Section 2, Kuang-Fu Road, Hsinchu, Taiwan 30013, Republic of China; Department of Materials Science and Engineering, National Tsing Hua University, No. 101, Section 2, Kuang-Fu Road, Hsinchu, Taiwan 30013, Republic of China; Department of Physics, The University of York, York YO10 5DD, UK; Department of Physics, The University of York, York YO10 5DD, UK; The Centre for High Resolution Neutron Scattering, National Institute of Standards and Technology, Gaithersburg, MD 20899, USA; Engineering and the Environment, University of Southampton, Southampton SO16 7QF, UK; Department of Engineering and System Science, National Tsing Hua University, No. 101, Section 2, Kuang-Fu Road, Hsinchu, Taiwan 30013, Republic of China; Department of Chemistry, National Chung Hsing University, No. 145 Xingda Rd., South Dist., Taichung City 402, Taiwan; Department of Materials Science and Engineering, National Tsing Hua University, No. 101, Section 2, Kuang-Fu Road, Hsinchu, Taiwan 30013, Republic of China

**Keywords:** core–shell, hyperthermia, magnetic interaction, magnetic resonance image, theranostics

## Abstract

The controlled size and surface treatment of magnetic nanoparticles (NPs) make one-stage combination feasible for enhanced magnetic resonance imaging (MRI) contrast and effective hyperthermia. However, superparamagnetic behavior, essential for avoiding the aggregation of magnetic NPs, substantially limits their performance. Here, a superparamagnetic core–shell structure is developed, which promotes the formation of vortex-like intraparticle magnetization structures in the remanent state, leading to reduced dipolar interactions between two neighboring NPs, while during an MRI scan, the presence of a DC magnetic field induces the formation of NP chains, introducing increased local inhomogeneous dipole fields that enhance relaxivity. The core–shell NPs also reveal an augmented anisotropy, due to exchange coupling to the high anisotropy core, which enhances the specific absorption rate. This in vivo tumor study reveals that the tumor cells can be clearly diagnosed during an MRI scan and the tumor size is substantially reduced through hyperthermia therapy by using the same FePt@iron oxide nanoparticles, realizing the concept of theranostics.

One of the current challenges in biomedical science is that of developing multifunctional materials with functionality for both ultrasensitive imaging and highly effective therapy. Magnetic nanoparticles (NPs) have been widely used as magnetic resonance imaging (MRI) contrast agents. When an external magnetic field is applied, the magnetic NPs that label cells cause a significant dephasing of protons due to the magnetic field inhomogeneity induced in the water molecules within the cells.^[1]^ Magnetic NPs also provide a promising application in cancer therapy through magnetic hyperthermia, which utilizes the heat generated by magnetic NPs under an alternating field to kill tumor cells.^[1–3]^ Magnetic NPs injected into the body for diagnosis could also be used in subsequent therapy. The concept of theranostics (a combination of diagnosis and therapy) would be highly advantageous to medicine as the same particles can be used for detection and for treatment of tumors.^[4]^ However, magnetic NPs serving as theranostic agents are still in an early stage of development.

Iron oxide nanoparticles (IONPs) have been considered a promising candidate for an MRI contrast and cancer treatment with hyperthermia due to their biocompatibility. The typical relaxivity and specific absorption rate (SAR) of iron oxide-based magnetic NPs range from 100 to 200 S^−1^ mMFe^−1^ and 100 to 500 W g^−1^, respectively, and still require further improvement to reduce the required dosage for clinical usage.^[1,2]^ Several approaches for optimizing the MRI contrast of IONPs have been demonstrated by varying their composition (saturation magnetization),^[5–9]^ surface treatment,^[8–13]^ or size.^[5–9]^ As for the SAR, power dissipation can be enhanced by tuning magnetic anisotropy, particle size, or saturation magnetization.^[14,15]^ To extend the functionality and overcome the limitations of single-component magnetic NPs, the magnetic core can be selectively modified to compliment the properties of the shell. The magnetic core–shell structure, which possesses tunable magnetic anisotropy and magnetization, has been demonstrated to achieve contrast-enhanced MRI^[16,17]^ or efficient heat induction.^[14]^ However, very limited reports so far demonstrate superparamagnetic NPs for highly effective theranostics, that is, enhanced *r*_2_ and SAR simultaneously.

Regarding MRI contrast, *r*_2_ relaxivity has been reported to depend on the properties of a single magnetic particle based on outer-sphere relaxation theory.^[18]^ However, during the MRI process, a strong DC magnetic field is applied to align the protons, and a small AC field is used to disturb their alignment, contributing to *T*_2_ relaxation due to local variations in precession rate. Therefore, magnetic NPs may interact with each other in the presence of magnetic fields and form various forms of clusters or assemblies. So far, no related investigations have addressed how magnetic interactions between NPs influence the formation of clusters or assemblies under magnetic fields. Likewise, the effect on *T*_2_-weighted MRI has not yet been discussed.

Here, we demonstrate the importance of the magnetic interaction between NPs for both the relaxivity and the SAR, which provides a new tuning knob for NP designs used for MRI and hyperthermia. By using core–shell superparamagnetic NPs composed of FePt core protected by a biocompatible cubic Fe_3_O_4_ shell, we can tune magnetic interactions among NPs as well as between the core and shell. The monodispersed FePt@IONPs are shown to simultaneously possess highly effective *T*_2_-shorting (*r*_2_ = 360 mM^−1^ s^−1^) and high-efficiency hyperthermia (SAR = 1.21 kW g^−1^), which enables therapy immediately after a diagnosis using the same NPs.

Cubic FePt@IONPs show a high *r*_2_ relaxivity of 360 mM^−1^ s^−1^ at 4.7 T, while the *r*_2_ relaxivity of cubic IONPs and Resovist are lower, at 129 and 194 mM^−1^ s^−1^ (Figure 1a). The FePt@IONP nanocubes also show significantly darkened phantom images, as shown in Figure 1b, which correspond to a strongly enhanced *T*_2_ contrast relative to the cubic IONPs and Resovist NPs. Resovist and our cubic IONPs show SAR values of 0.39 and 0.92 kW g^−1^, respectively, while FePt@IONPs exhibits a value of 1.21 kW g^−1^ (Figure 1c). (See Section S1, Supporting Information, for details of the SAR calculations of NPs.) For the cancer hyperthermia treatment, the temperature needs to be increased to 42 °C. Because of the high SAR, the required dose and exposure time to reach 42 °C in the in vitro experiment are only 0.1 mg mL^−1^ of FePt@IONPs and 100 s, respectively, measured with an AC magnetic field of 18.8 kA m^−1^ at 630 kHz and with dispersion volume of 0.2 mL. For the in vivo theranostic demonstrations presented here, MRI was acquired with a quadrature surface coil using a spin echo sequence with DC magnetic field, and magnetic hyperthermia was applied with an AC magnetic field provided by a magnetic induction local coil with a diameter of 3 cm^[19]^ (Figure 1d). Theranostic application of core–shell FePt@IONPs was carried out in KB human cervical cancer cells xenografted mice model. FePt@IONPs were intravenously injected into mice at 0.1 mg mL^−1^ with the dispersion volume of 0.2 mL; 24 h later, the mice were placed in a 3 T MRI head coil and subsequently under the magnetic induction local coil for magnetic hyperthermia. The tumor MR contrast ability of FePt@IONP is about 1.5 times higher than that of Resovist (Figure 1e). Note that the different mice may exhibit different background MR signals in tumor areas. In our tumor MR contrast, we compare the contrast in tumor area with background in the same mice so that we can do fair comparison for MRI contrast with different contrast agents.^[20]^ We also compare MR images taken at kidney and liver before and after intravenous injection of FePt@IONPs, shown in Figure S1 (Supporting Information). We can clearly observe the enhanced contrast after the injection of FePt@IONP. For magnetic hyperthermia, the tumor area was treated with an AC magnetic field of 18.8 kA m^−1^ at 630 kHz for 10 min and the tumor size was monitored for up to half a month. Significant delay of tumor growth was observed in FePt@IONP hyperthermia group at day 15 whereas Resovist hyperthermia group revealed no inhibition of tumor growth compared with untreated control group (neither NPs nor hyperthermia treatment) (Figure 1f). Note that the tumor volume still increases for the FePt@IONP control sample (with FePt@IONP but without hyperthermia), which clearly indicates that FePt@IONPs show efficient magnetic hyperthermia treatment of the cancer due to their high SAR. Furthermore, the increased tumor volume in the FePt@IONP control sample also suggests that cytotoxicity of FePt@IONP is not severe. Clear MRI images of NPs can accurately locate the tumor region where the same NPs can be used for highly effective hyperthermia to realize the theranostic modality of precise diagnosis and treatment.

To extend the functionality and overcome the limitations of single-component magnetic NPs, the magnetic core can be selectively modified to compliment the properties of the shell. Figure 2a shows the transmission electron microscopy (TEM) image of Fe_3_O_4_ nanocubes. The average edge length of Fe_3_O_4_ cubes is 16.1 ± 0.9 nm and that of the bi-magnetic core–shell FePt@IONPs with FePt core and cubic shell Fe_3_O_4_ is 14.7 ± 1.1 nm (Figure 2b). The disordered FePt NPs are spherical, exhibiting superparamagnetic behavior due to their small size of about 4.1 nm. The high-quality single-crystalline structure of the nanocubes is shown in Figure 2c. The FePt core obviously embedded inside the cubes was demonstrated by aligning a tilt series in 3D TEM tomography (Figure 2c). The X-ray diffraction (XRD) patterns of the nanocube assembly on Si substrates indicate that our IONP structure corresponds to the Fe_3_O_4_ lattice (Figure S2a–c, Supporting Information). The X-ray photoelectron spectra (XPS) of IONPs and FePt@IONPs also show no satellite peak between the peaks of Fe 2p_3/2_ and Fe 2p_1/2_, indicating that these nanocubes are consistent with the Fe_3_O_4_ component (Figure S2e,f, Supporting Information).

Before discussing the mechanism of superior performance of the FePt@INOPs in both MRI contrast and hyperthermia, we first evaluated the cytotoxicity and stability of FePt@INOPs dispersed in water. To demonstrate the effects of magnetic interaction of NPs on MRI contrast, we specifically used a surfactant with short-chain molecules, CTAB (hexadecylcetyltrimethylammonium bromide, FW: 364.45), a well-known surfactant for dispersing and stabilization of NPs. On the other hand, CTAB-stabilized NPs used for bioapplications may cast doubts about their cytotoxicity. Therefore, we also used mPEG (methoxypolyethylene glycol, FW: 350) as a surfactant. The mPEG-coated IONP is widely used for bioapplication and reveals no cytotoxicity.^[21]^ The selected mPEG possesses similar molecular weight to CTAB and its short-chain molecule does not significantly change the hydrodynamic diameter, therefore, mPEG-coated NPs may provide the similar magnetic interactions among NPs to CTAB-coated NPs. The enhancements of *r*_2_ and SAR by using mPEG-coated FePt@IONPs (Figure S3, Supporting Information) are similar to those shown in Figure 1, by using CTAB-coated FePt@IONPs. Figures S4 and S5 (Supporting Information) show 3-(4,5-dimethylthiazol-2-yl)-2,5-diphenyltetrazolium bromide (MTT) assay for KB cells and hematoxylin and eosin (H&E) staining of major organs after the magnetic hyperthermia treatment, respectively. Both results clearly demonstrate that mPEG-coated FePt@IONPs have no toxic reaction for KB cells and organs. In addition, dynamic light scattering (DLS) data reveal that the particle size for monodisperse FePt@IONP and IONP does not change significantly after 24 h (Figure S6, Supporting Information), indicating no aggregation occurs. All results clearly demonstrate that our FePt@IONP can be directly applied to MRI and hyperthermia without concerns of cytotoxicity and stability.

The hysteresis loops for nanocubes in water (Figure 3a) reveal that both cubic IONPs and FePt@IONPs exhibit superparamagnetic behavior at room temperature. The theoretical saturation magnetizations of bulk Fe_3_O_4_ and FePt are 471 and 1140 emu cc^−1^, respectively. The FePt core does not contribute significantly to the magnetic moment as it accounts for merely 1% of the volume of the magnetic structure. When the proton diffusion length is not negligible with respect to the size of NPs, the MRI relaxivity can be described in the motional averaging regime of protons. The relaxivity is expected to be directly proportional to the NP size squared and to the saturation magnetization.^[22,23]^ Also, the SAR value is expected to be linearly dependent on the saturation magnetization for superparamagnetic NPs.^[1]^ Since our cubic IONPs and FePt@IONPs have similar sizes and saturation magnetization, the enhanced contrast and heating efficiency of FePt@IONPs in comparison to IONPs cannot be attributed to these two factors. A slight increase of coercivity for FePt@IONPs at a low temperature can be attributed to the interaction between the magnetically soft IONP shell and relatively hard FePt core (Figure S7, Supporting Information). In addition, hysteresis loops show that the initial susceptibility of cubic IONPs is higher than that of FePt@IONPs. The frequency-dependent susceptibilities (Figure 3b) also reveal higher susceptibility for IONPs. It should be noted that the IONPs show a broader peak of the imaginary part of the susceptibility compared to FePt@IONPs. The fits of the susceptibility for polydisperse model of IONPs and monodisperse model of FePt@IONPs are presented in Figure S8 (Supporting Information),^[24–27]^ which indicate that the magnetic interaction between IONPs in solution is stronger than that between FePt@IONPs.

To further quantify the particle interaction of structurally uniform magnetite NPs,^[28,29]^ we performed small-angle neutron scattering (SANS) at the NIST Center for Neutron Research on beam lines NG3 and NG7. The nanocubic IONPs and FePt@IONPs were measured in a D_2_O solution at the concentration of 5 mg mL^−1^. The neutron scattering from D_2_O is similar to that of Fe_3_O_4_, thereby largely masking the structural scattering of the Fe_3_O_4_ while retaining its full magnetic scattering intensity. The cubic FePt@IONPs exhibit a *Q*^−2.5^ Porod slope, as shown in Figure 3c, consistent with the presence of surfactant with a slope between that of mass fractal and surface fractal scattering.^[28,29]^ The cubic IONPs display the same Porod slope plus the addition of a shoulder which can be fitted using a cluster model of size 88 nm (or roughly 5–6 nanocubes). Given that the cubic FePt@IONPs exhibit no structural scattering features associated with the nanocube shape, the IONP cluster scattering appears to be magnetic in origin. The SANS data indicate that the IONPs tend to cluster together, while the FePt@IONPs nanocubes pack less tightly and do not exhibit aggregation when solvated. Consequently, the IONPs form interparticle magnetic domains, while the magnetic domains of FePt@IONP nanocubes are limited in size to a single NP and exhibit no apparent magnetic coupling. Solvated IONPs consistently exhibit magnetic correlations, while FePt@IONPs do not. According to the SANS results, the primary function of the FePt is to disrupt the formation of the long-range magnetic dipolar interaction at zero field thereby reducing clustering in FePt@IONP.

Furthermore, using electron holography to observe interference fringe patterns allows a phase shift of the high-energy electron wave transmitted through the specimen to be measured by high-resolution TEM.^[30–32]^ The electron holography of magnetic structures for FePt@IONPs and IONPs in dried samples demonstrates the natural magnetic vector fields within and between the particles, as shown in Figure 4a, b. The observed flux-closure domain of FePt@IONPs prevents the NPs from interacting with each other. The domain structure of nanocubes was simulated by using micromagnetic calculations (OOMMF code),^[32]^ as shown in Figure S9 (Supporting Information). The magnetic induction flux lines in the two IONPs present an induction state characteristic of interacting nanocubes, but the flux closure state exhibited by the FePt@IONPs indicates a magnetic vortex configuration. The flux closure state of FePt@IONPs may explain a diversity of observations, including the suppressed long-range magnetic dipolar interaction in solution, susceptibility measurements, and small-angle neutron scattering. The suppressed magnetic interaction in FePt@IONPs also helps prevent NP aggregation and cluster formation, consistent with our in vivo observation that mice survival rate is increased with FePt@IONPs due to the reduced detrimental effects on blood circulation.

During the MRI measurements, a strong magnetic DC field and a small orthogonal AC pulse field are applied, which may alter the NP configuration. To understand how the magnetic field influences the formation of magnetic clusters or assemblies, we deposited a solution containing NPs on a TEM grid. During the drying process, we applied magnetic fields. When a DC field of 0.47 T was applied, the IONPs yield bundles of IONP chains aligned with the DC field direction (Figure 4c). The long chains are broken down into separate small clusters by simultaneously applying an orthogonal AC pulse field of 0.5 mT at 20 MHz and a DC field of 0.47 T. The separated clusters are still aligned with the DC field (Figure 4d). On the other hand, the magnetically aligned FePt@IONP chains under a DC field (Figure 4e) are essentially unchanged by simultaneously applying AC pulse fields (Figure 4f). The evolutions of chains of IONPs and FePt@IONPs dispersed in water with a concentration of 0.5 × 10^−3^ M under DC and pulse AC fields applied were recorded in situ using an optical microscope and are shown in Movies S1 and S2 (Supporting Information). The recording clearly demonstrates that the FePt cores seem to be strongly aligned by the DC field so that the FePt@IONPs chains cannot be dissociated with pulse AC fields. The effects of DC field on the assembly of IONPs and FePt@IONPs are also shown in Figure S10 (Supporting Information). When fields are off, both IONPs and FePt@IONPs chains are well dispersed again in the solution (Figure S10, Supporting Information). Furthermore, the AC susceptibility spectra for both NPs in solution are unchanged after removing magnetic fields, which confirms the reversible assembly process from monodispersed NP (no field) to chains (in DC field) (Figure S11, Supporting Information).

To understand how the interaction between NPs affects relaxation rate *R*_2_ (*R*_2_ = 1/*T*_2_) in MRI measurement, we performed a Monte Carlo simulation as described in refs.^[19,20]^ and the Experimental Section. To compare experiments, we investigated the role of the local configuration by simulating the MRI of discontinuous chains for the IONP sample and continuous chains for the FePt@IONP sample. Each discontinuous or continuous (6 × 2 × 2) chain contains 24 particles. In addition, for comparison, we considered the case of single randomly distributed particles. For each type of configuration, the system was constructed using ≈1500 magnetic NPs. The relaxation rate as a function of concentration (packing density) for each configuration type is illustrated in Figure 5a. The *r*_2_ relaxivity of the FePt@IONPs continuous chain is about 2.15 times higher than flocculated clusters of IONPs, which compares well to the experimental observation of 2.8 times (Figure 1a). The relaxation curves for the large structures (as experimentally illustrated in Figure 4) were also calculated to examine the role of the NP configurations at this scale (Figure S12, Supporting Information).

The simulation corroborates the experimental data, showing the influence of spatial configuration on magnetic particle properties. Importantly, it is demonstrated that the performance of magnetic NPs in MRI does not depend just on macroscopic properties such as packing density (average concentration), but also on microscopic properties (local concentration or local configuration/arrangement). The *R*_2_ depends on the dipole field acting on each proton. This is nonuniform and strongly influenced by the geometry/position of the magnetic NPs as illustrated in Figure 5b, c. Here, we show the dipole map of a section of the sample, which indicates the location and configuration of NPs. The dipole field map varies locally throughout the sample. This is supported by the dipole field histogram computed over the entire sample (Figure 5d), which highlights the influence of the NP configuration in enhancing the spatial distribution of dipolar fields. This has an important effect on the MRI performance as the diffusing protons will experience different dipolar fields depending on the spatial location. If we analyze the tail of the dipole field distribution (related to large field and therefore fast dephasing, Figure 5e) we can see that there is a direct correlation of the distribution tail with the *R*_2_ values. The NP chain has a systematically larger volume with a stronger dipole field compared to the discontinuous chains (discrete flocculated clusters). This corresponds to a faster dephasing of the protons and therefore an enhancement of the MRI performance (i.e., a larger *R*_2_). As all the systems investigated here are identical, with the exception of the spatial configuration, we can conclude that the details of NP configuration have an important role to play in determining the MRI performance.

With the understanding of magnetic interaction effects on the relaxation and MRI contrast, we would like to further illustrate the uniqueness of our core–shell structures. The presence of superparamagnetic FePt core plays an important role on the magnetic interaction among NPs. At zero field, the existence of a FePt core leads to the formation of closure domains within FePt@IONPs, which disrupts the formation of the long-range dipolar interaction. The energy cost of forming a vortex is the exchange energy associated with a point divergence of the magnetization at the center. Applying a field stabilizes the magnetization of FePt and makes the vortex structure less energetically feasible, leading to the observed chain formation in an applied field. The reduced dipolar interaction in zero field helps the circulation of NPs in the bloodstream and enhances SAR. On the other hand, under a strong DC field, the FePt core is strongly aligned by the DC field such that the NPs chains are not disturbed by the applied AC pulse field. The chain configuration formed by magnetic fields reveals a stronger dipolar field around the chain, which corresponds to a faster dephasing of the proton and therefore an enhancement of the MRI performance. We would like to emphasize that our approach can be combined with other methods to further increase relaxivity. For example, by increasing the particle size (17.8 nm) of superparamagnetic core–shell structure, we can further improve the relaxivity to 411.3 mM^−1^ s^−1^, as shown in Figure S13 (Supporting Information). Our approach shows great potential to reach even higher *r*_2_ by further optimization. Thus, highly effective multifunctional NPs for bioapplications can be successfully realized by designing core–shell structures to tune magnetic interactions among NPs.

For hyperthermia treatment, cubic NPs have been reported to have a higher SAR than spherical ones due to increased anisotropy.^[33]^ The magnetic core–shell structure with exchange coupling between a magnetically hard core and soft shell can further enhance anisotropy and thus the SAR.^[14]^ In addition to anisotropy, the simulations reveal that reduced magnetic dipolar interaction among NPs can raise the SAR.^[34]^ Both susceptibility spectra (Figure 3b) and small-angle neutron scattering spectra (Figure 3c) show that the FePt@IONP suspension have greatly reduced magnetic dipolar interactions between NPs in zero field compared to IONPs, which may also contribute to the enhanced SAR.

In summary, we demonstrate that superparamagnetic cube FePt@IONPs core–shell NPs are suitable to be used as theranostic agents because of their higher relaxivity and SAR value. We reveal that the magnetic NP interaction not only influences the *T*_2_-weighted image but also the SAR. The FePt core is essential for forming the specific flux-closure domain in the core–shell structure. The cubic FePt@IONP core–shell has several prominent advantages. First, the magnetic core–shell structure, with lower magnetic dipole–dipole interactions among the particles, can be designed to stabilize magnetic NPs against aggregation caused by magnetic interactions in the remanent state. Second, the FePt core may be exchange-coupled to the IONP shell, which significantly enhances the SAR. Furthermore, the presence of the FePt core maintains chain-like assemblies during AC field pulses in MRI measurement, which essentially enhances *R*_2_. With the ability to tune magnetic interactions between NPs, the design of multifunctional cubic FePt@IONP can be devoted to improving the resolution of the MRI image and effective hyperthermia for theranostic applications.

## Experimental Section

### Synthesis of FePt Cores:

Synthesis of FePt NPs was carried out by using standard Schlenk line techniques under Ar atmosphere. In a typical synthesis process, Pt(acac)_3_ (0.25 mmol), Fe(CO)_5_ (0.75 mmol), 1,2-hexadecanediol (2 mmol), oleic acid (1.2 mmol), and oleylamine (1.2 mmol) were mixed and dissolved in benzyl ether (10 mL). The mixed solution was heated to 290 °C with a heating rate of 5 °C min^−1^. The temperature was kept at 290 °C for 30 min. Then the solution was naturally cooled to room temperature. The NPs were washed by ethanol and redispersed in hexane several times. The final product was stored in hexane.

### Synthesis of Cubic Fe_3_O_4_ and FePt@IONP:

The details of synthesis of cubic Fe_3_O_4_ can be found in ref. [35] and the similar approach was used for the synthesis of the shell of FePt@IONPs. The synthesis of the shaped FePt@IONP core/shell nanocubes involved two steps, including the synthesis of a FePt core and the overgrowth of a shaped Fe_3_O_4_ shell. The prepared FePt NPs were mixed with oleic acid (0.3 mmol), oleylamine (0.3 mmol), and 1,2-tetradecanediol (5 mmol) and were dissolved in benzyl ether (15 mL) which served as the reaction solution. For the precursor solution, Fe(acac)_3_ (1 mmol) was dissolved in benzyl ether (5 mL). The reaction solution was dewatered at 120 °C for 1 h and was then heated to the reflux temperature of 290 °C. Then, the precursor solution was injected into the hot reaction solution at 290 °C with an injection rate of 10 mL h^−1^. The fluctuation of the reaction temperature was less than 5 °C during the injection process. After the reaction was finished, the NPs were washed in ethanol and redispersed in hexane. Both Fe_3_O_4_ NPs and FePt@IONP were modified with short-chain surfactants (such as CTAB or mPEG) to be dispersed in water.

### Synthesis of CTAB-FePt@IONP:

The core–shell FePt@IONPs were then dried under vacuum and added into an aqueous solution containing 0.1 m CTAB (FW: 364.45). After 10 min sonication, the core–shell FePt@ IONP NPs were coated by CTAB and formed stable NP dispersion in water.

### Synthesis of mPEG-FePt@IONP:

The dopamine-based surfactant to replace oleylamine around the iron oxide nanoparticles as reported in ref. [36] was used. To functionalize the FePt@IONP nanoparticles with mPEG, trichloro-*s*-triazine (TsT) (22 mg) was first used to react with mPEG (average mol wt: 350) (200 mg) at room temperature in anhydrous benzene (20 mL) to form TsT-mPEG. Then, TsT-mPEG (20 mg) reacts with dopamine hydrochloride (20 mg) in 1,4-dioxane solvent, forming compound. The catecol unit in dopamine-based molecule was used to replace oleylamine around the iron oxide NPs, forming stable NP dispersions in water.

## Supplementary Material

suppl

## Figures and Tables

**Figure 1.**
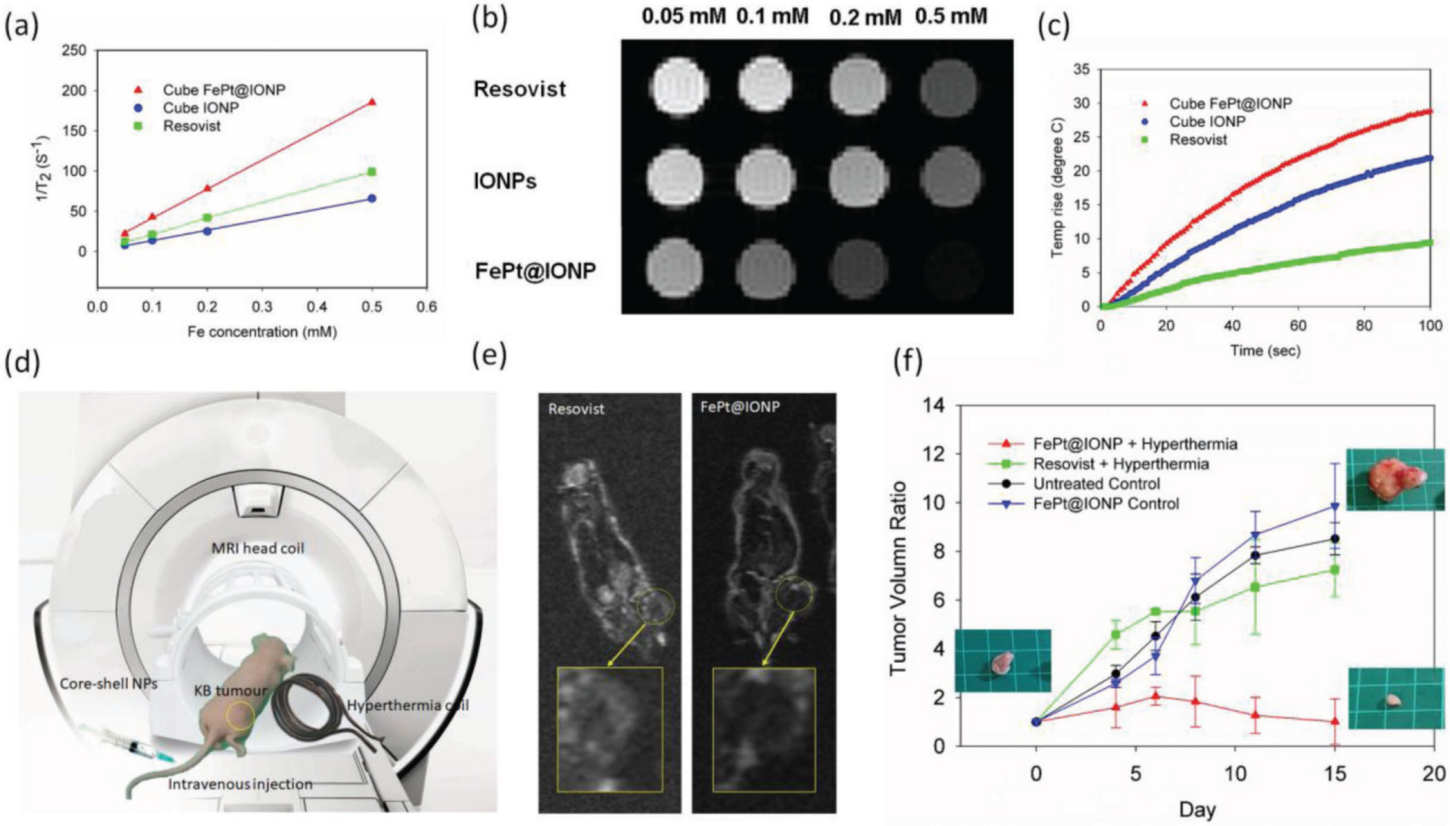
a–c) The relaxivity and hyperthermia measurement of NPs. a) The variation of 1/*T*_2_ with Fe concentration of cubic FePt@IONPs (red triangle line), Resovist (green square line), and cubic IONPs (blue circle line) measured at 4.7 T. The *r*_2_ relaxivities (mM^−1^ s^−1^) were obtained from fitting the slope of each sample. b) *T*_2_-weighted images of Resovist, cubic IONPs, and cubic FePt@IONPs with the same Fe concentration, measured at 4.7 T. c) Measurement of heat generation of the NPs, carried out by using an AC magnetic field produced from radiofrequency heating machine. The rate of the temperature increase was 0.29, 0.22, and 0.04 °C s^−1^ for FePt@IONP, IONP and Resovist, respectively. d) In vivo thernanostic performance of NPs, schematic picture shows real-time MRI-controlled magnetic hyperthermia system for tumor treatment, e) *T*_2_*-weighted MR images of KB tumor of a mouse with Resovist (left) and FePt@IONP (right). f) Plot of tumor volume ratio (tumor volume/initial tumor volume) versus days after treatment with FePt@IONP hyperthermia, Resovist hyperthermia, untreated control, and FePt@IONP control.

**Figure 2.**
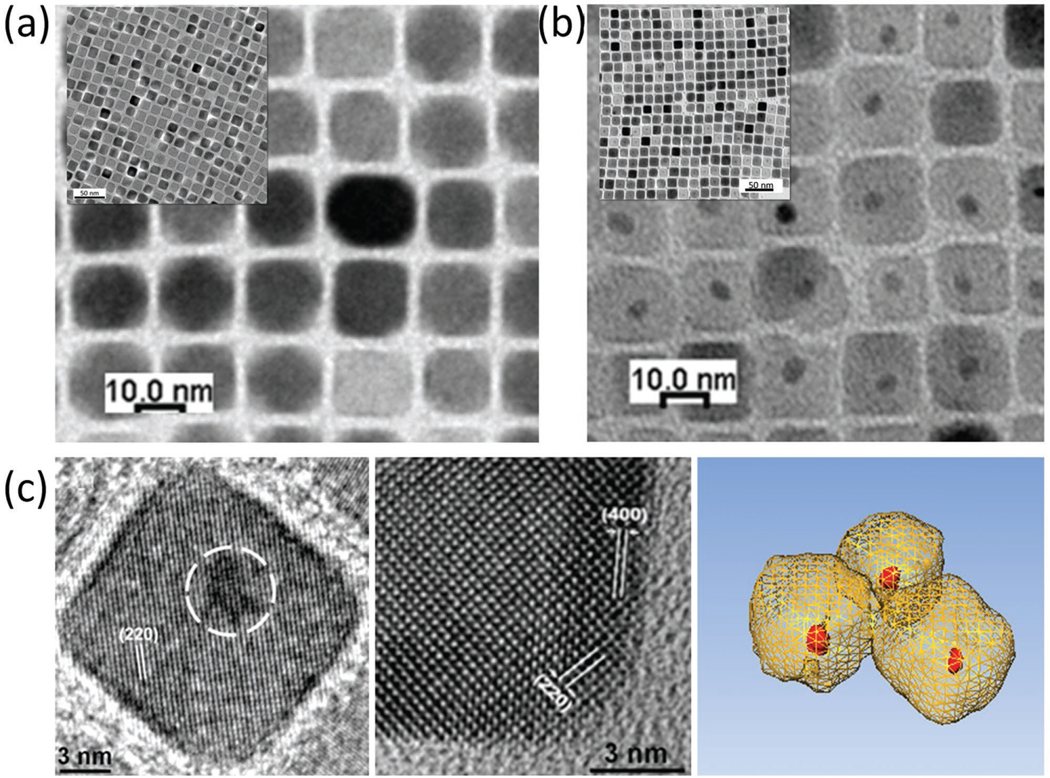
Structural and microstructural analyses of IONPs and FePt@IONPs. a) TEM image of cubic IONPs. b) TEM image of cubic FePt@IONPs. c) (Left) The core–shell NPs with FePt core and Fe_3_O_4_ cubic shell are further demonstrated by the HRTEM images (the dashed circle indicates the position of FePt core). (Middle) The lattice fringes correspond to {220} lattice planes of IONPs while the facet is {100}. (Right) 3D TEM tomography of the FePt@IONPs core–shell structure.

**Figure 3.**
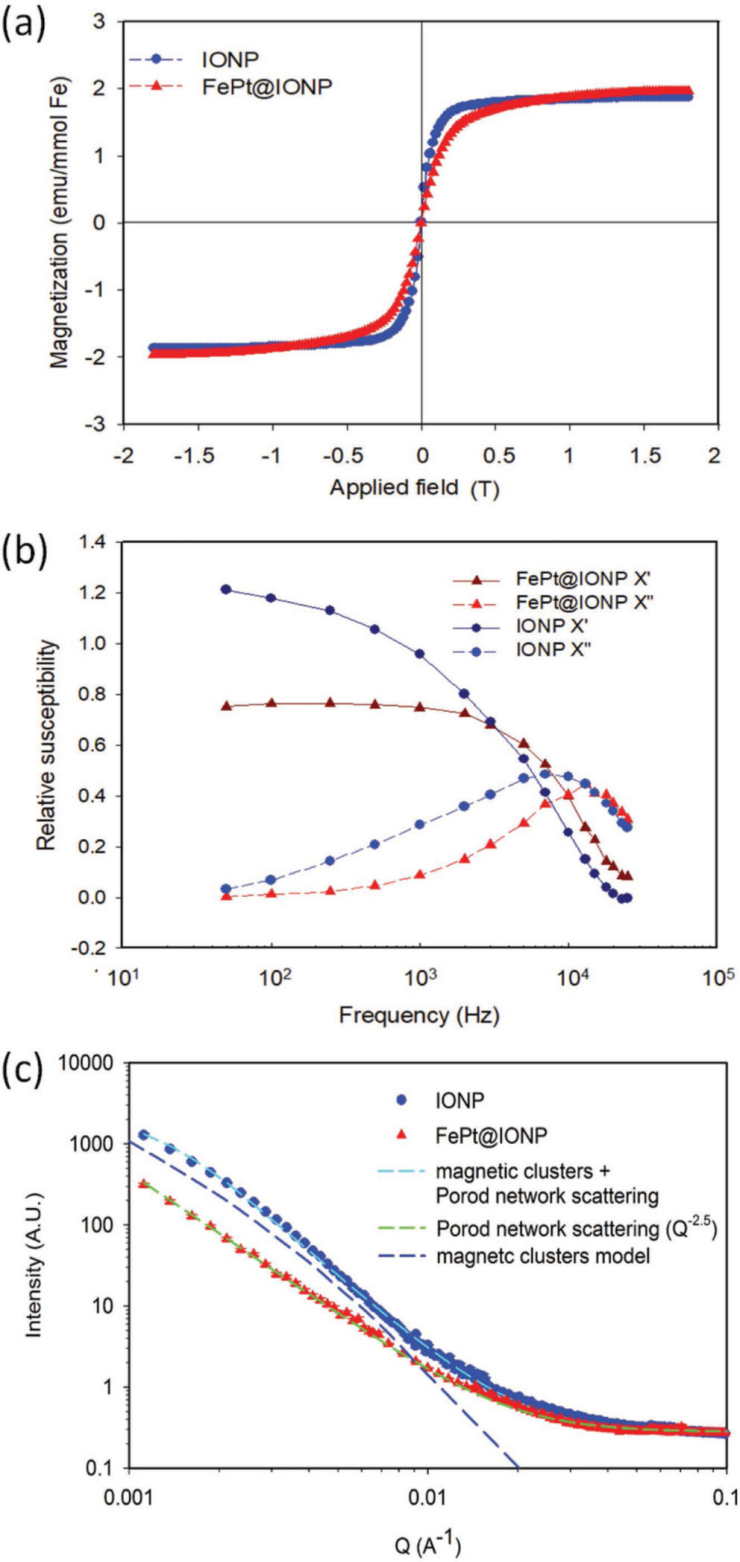
Magnetic properties and magnetic structural scattering of NPs. a) Magnetic hysteresis loops of FePt@IONPs and IONPs dispersed in water with the concentration of 1 mgFe mL^−1^. b) The real and imaginary parts of magnetic susceptibility frequency spectra of FePt@IONPs and IONPs dispersed in water with the concentration of 1 mgFe mL^−1^. c) Small-angle neutron scattering fitted by the Porod network scattering and magnetic clusters model. Solvated cubic IONPs (blue circle line) reveal long-range magnetic scattering that is absent in cubic FePt@IONPs (red triangle line).

**Figure 4.**
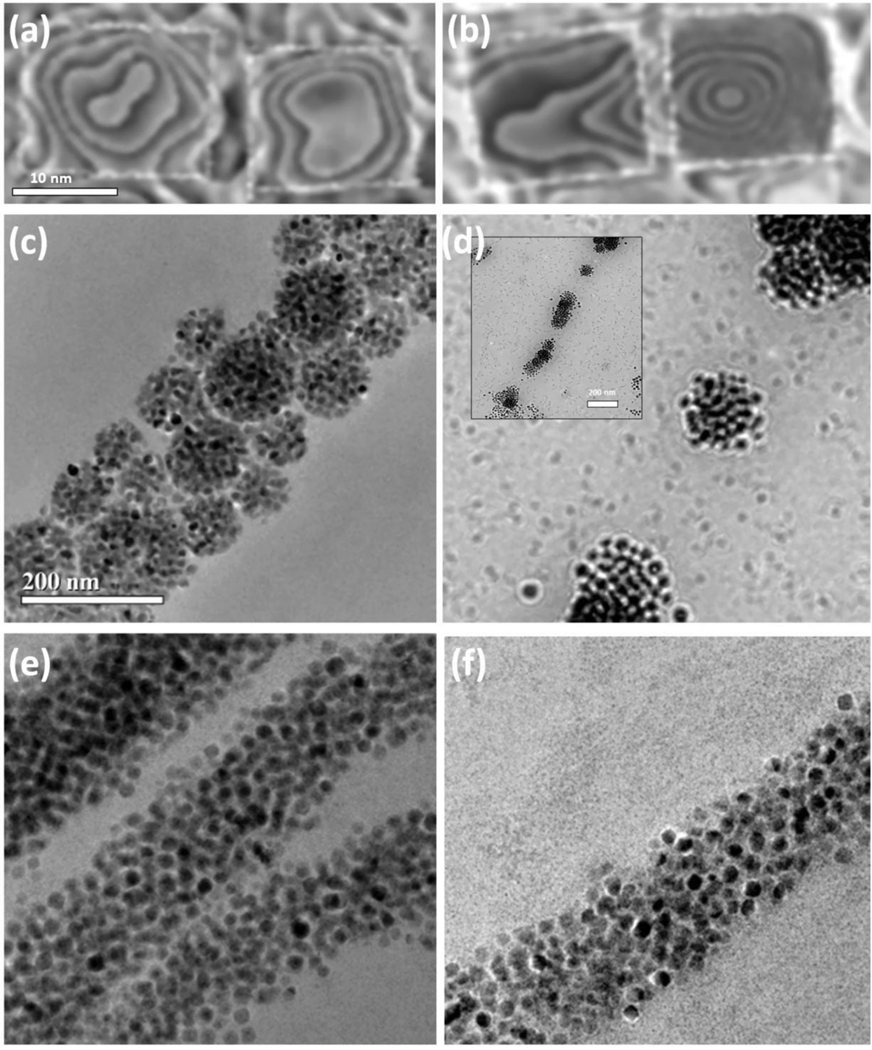
Electron holography and TEM analyses of NPs. a,b) Magnetic induction maps recorded using off-axis electron holography at the remanent state for cubic FePt@IONPs (a) and cubic IONPs (b) (the white dotted lines are added to indicate the location of NPs). c–f) TEM images, and the inset in (d) shows a large-scale image, for NPs with a Fe concentration of 0.5 × 10^−3^ M. c) IONPs under a DC magnetic field of 0.47 T and d) IONPs under the DC magnetic field of 0.47 T and an orthogonal AC field of 0.5 mT at 20 MHz. e) FePt@IONPs under a DC magnetic field of 0.47 T and f) FePt@IONPs under a DC magnetic field of 0.47 T and an orthogonal AC field of 0.5 mT at 20 MHz.

**Figure 5.**
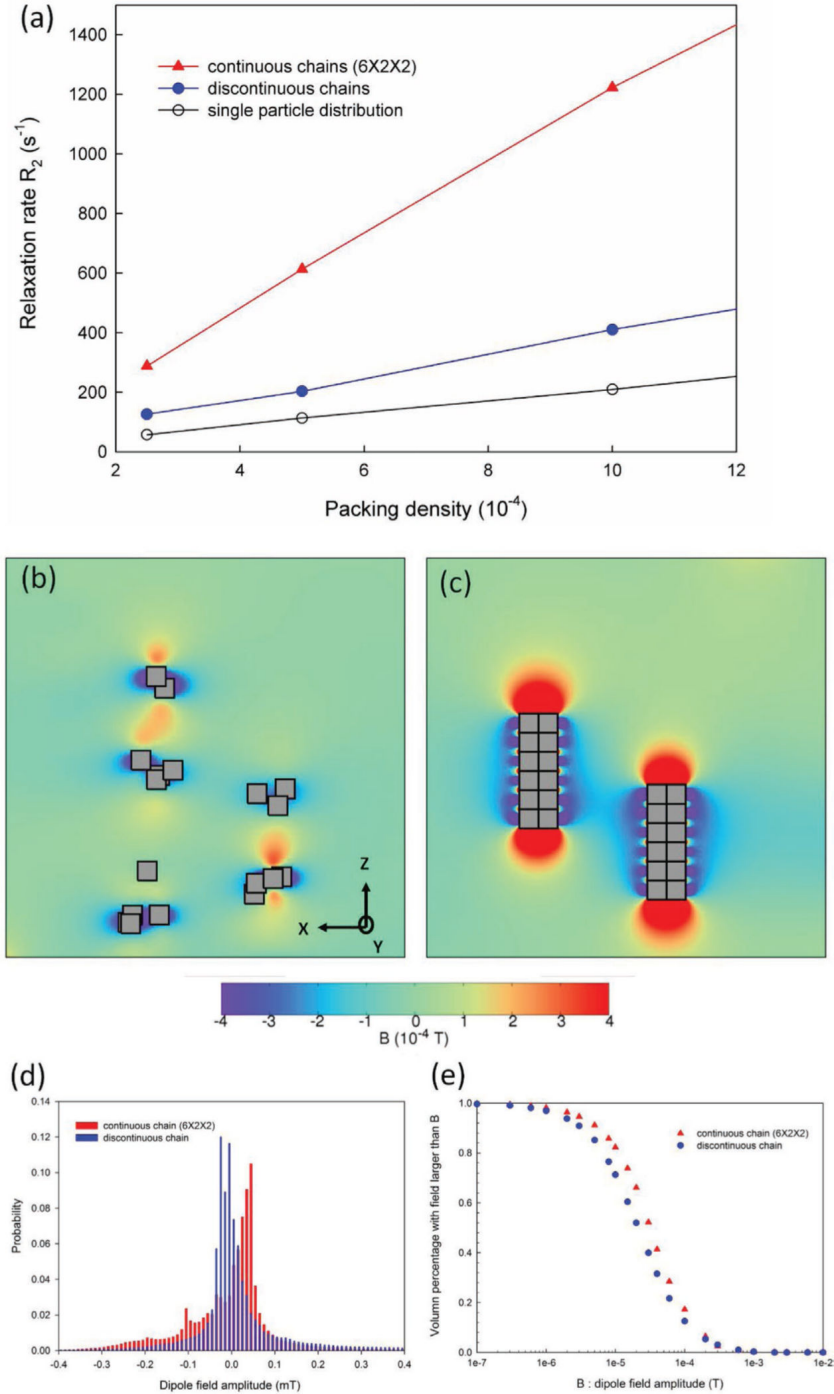
*R*_2_ results from Monte Carlo simulation. a) The dependence of *R*_2_ on the packing density for two configurations of NPs: the continuous chains (red triangle) and the aligned discrete clusters (discontinuous chain: blue circle). For reference we also consider the case where the NPs are separated (single particle: open circle). b,c) Color maps of the dipole field in systems of discontinuous chains (*X–Z* plane) (b) and continuous chains (6 × 2 × 2) (c). The number of particle per cluster for discontinuous chains is 6 in the 3D structure. d) Statistics of the dipole field amplitude for continuous and discontinuous chains. e) Statistics of volume percentage with field larger than a given value. This corresponds to the tail of the dipole field distribution in the sample volume, which is responsible for the fast dephasing. The continuous chain has a larger field overall, leading to a larger *R*_2_.
