# Supplementary material for: Magnetic Interaction of Multifunctional Core–Shell Nanoparticles for Highly Effective Theranostics
Source: Adv Mater. Author manuscript; Available in PMC 2024 Jul 29. (PMC11284570; doi:10.1002/adma.201802444)
Supplement: suppl [file NIHMS1613506-supplement-suppl.pdf]

Copyright WILEY-VCH Verlag GmbH & Co. KGaA, 69469 Weinheim, Germany, 2018.

ADVANCED MATERIALS

Supporting Information

for *Adv. Mater.*, DOI: 10.1002/adma.201802444

Magnetic Interaction of Multifunctional Core–Shell Nanoparticles for Highly Effective Theranostics

*Ming-Da Yang, Chien-Hsin Ho, Sergiu Ruta, Roy Chantrell, Kathryn Krycka, Ondrej Hovorka, Fu-Rong Chen, Ping-Shan Lai, and Chih-Huang Lai**

Supporting Information

Magnetic Interaction of Multifunctional Core–Shell Nanoparticles for Highly Effective Theranostics

*Ming-Da Yang, Chien-Hsin Ho, Sergiu Ruta, Roy Chantrell, Kathryn Krycka, Ondrej Hovorka, Fu-Rong Chen, Ping-Shan Lai, and Chih-Huang Lai**

S1: SAR calculations of IONPs and FePt@IONPs.

The experimental setup for magnetically induced hyperthermia included a copper coil cooled with circulating water, and a resonant RLC circuit producing an AC magnetic field up to 18.8 kA/m at 630 kHz, which could increase the temperature of magnetic nanoparticles in water. By using the optical fiber thermometer to probe temperature in the center of the sample, the specific absorption rate (SAR) of superparamagnetic material is deduced from the initial linear rise of the plot of temperature versus time, ΔT/Δt (Figure 1c in the manuscript) and the heat capacity of the sample, normalized to the mass of magnetic material. The nanoparticle concentration in water was 1 mg/ml. SAR can be expressed as Equation S1 [1, 2, 3]:

$$SAR = C\frac{\Delta T}{\Delta t} \qquad \text{(S1)}$$

where C is the volumetric specific heat capacity of water, 4185 J/(kg・K). From the experimental data, commercially available Fe_3O_4 nanoparticles (Resovist) and our cubic IONPs show SAR values of 0.39 kW/g and 0.92 kW/g, respectively, while FePt@IONPs exhibits a value of 1.21 kW/g.

S2: T2*-weighted MR images of FePt@IONPs.

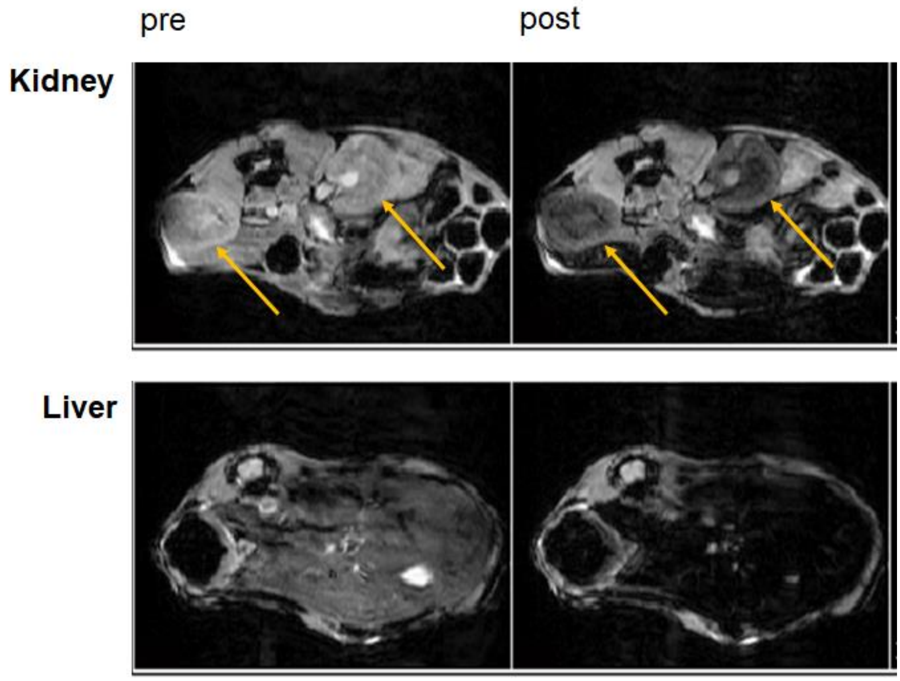

Figure S1. MR images for kidney and Liver of mice before and after intravenous injection of FePt@IONP.

The images with a body-adapted mould demonstrate a significantly improved MRI contrast in the kidneys and livers of mice between Pre- and Post-injection (shown in Figure S1).

S3: Structural analyses of IONPs and FePt@IONPs.

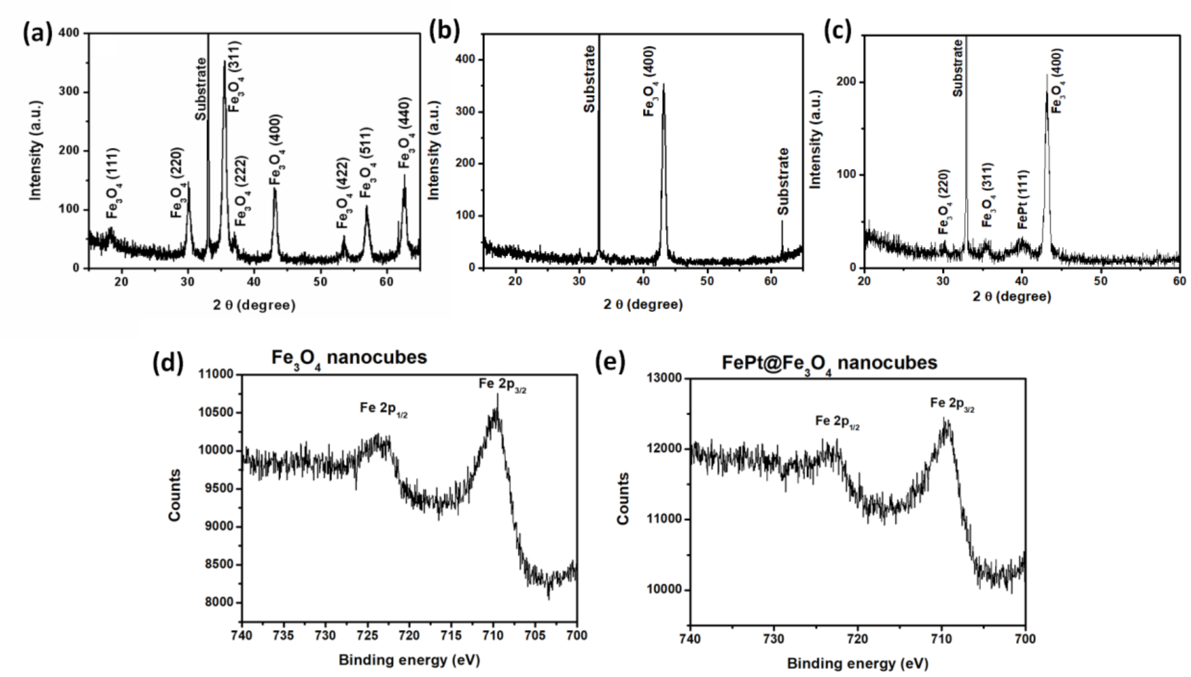

Figure S2. The XRD data of (a) IONPs with randomly packed, (b) IONPs with cube surface parallel to the substrate and (c) FePt@IONPs with cube surface parallel to the substrate, X-ray photoelectron spectrum (XPS) of (d) Fe_3O_4 nanocubes, and (e) FePt@Fe_3O_4 nanocubes

X-ray diffraction (XRD) patterns were detected by using Shimadzu XRD 6000 with Cu Kα radiation. The XRD samples were prepared by dropping the nanoparticle dispersion on Si substrates with a controlled solvent evaporating rate. After the solvent was evaporated, the dried nanoparticles on Si substrates were used for XRD measurement. With the varied evaporation rate of solvent, the nanocubes can be either randomly packed, or packed with the cube surface parallel to the substrate, that is, (400)-preferred orientations. The XRD data of IONPs with randomly packed (a), IONPs with cube surface parallel to the substrate (b) and FePt@IONP with cube surface parallel to the substrate (c) are shown in **Figure S2**. The XPS measurements were collected using synchrotron-based light source at the National Synchrotron Radiation Research Center (NSRRC) in Hsinchu, Taiwan. This method was chosen because synchrotron radiation provides high-intensity light of well-defined characteristics with a continuous spectrum ranging from infrared to X-ray. No satellite peaks

between the peaks of Fe $2p_{3/2}$ and Fe $2p_{1/2}$ were observed for Fe_3O_4 and FePt@Fe_3O_4 nanocubes, as shown in Figure S2 (d) and (e), indicating that these nanocubes were consistent with the Fe_3O_4 component.

S4: Surfactant effect on the r_2 and SAR of FePt@IONP NPs

(a) (b)

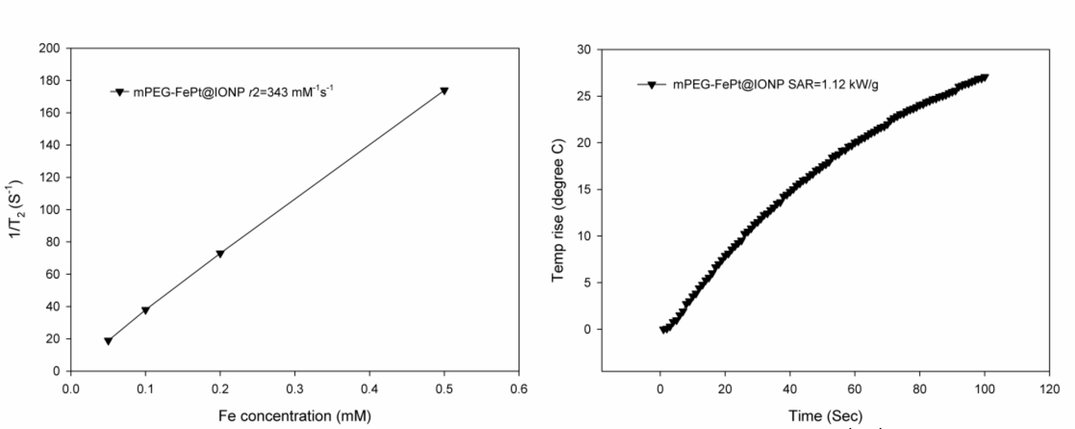

Figure S3 (a) The r_2 relaxivity of mPEG-FePt@IONP is 343 $mM^{-1}s^{-1}$. (b) The rates of temperature increase is 0.268℃/s for mPEG-FePt@IONP.

To improve bio-compatability of NPs, we change the surfactant CTAB to mPEG. The reuslts shown in Figure S3 reveal that no significant difference between CTAB and mPEG coated FePt@IONPs on the enhancement of MRI (r_2) and hyperthermia (SAR). The r_2 relaxivities of CTAB-FePt@IONP and mPEG-FePt@IONP are 360 and 343 $mM^{-1}s^{-1}$, respectively. The rates of temperature increase are 0.290℃/s and 0.268℃/s for CTAB-FePt@IONP and mPEG-FePt@IONP, respectively. The corresponding SAR values are 1.21 KW/g and 1.12 KW/g, respectively.

S5: Cytotoxicity evaluation of FePt@IONP NPs

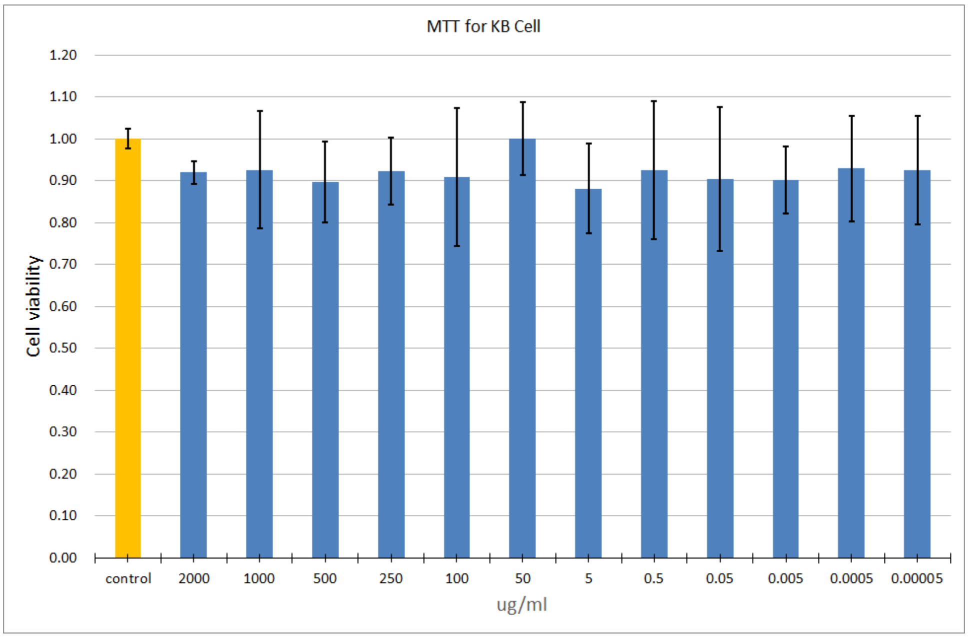

Figure S4. Effect of different concentration of mPEG-coated FePt@IONPs on cell viability

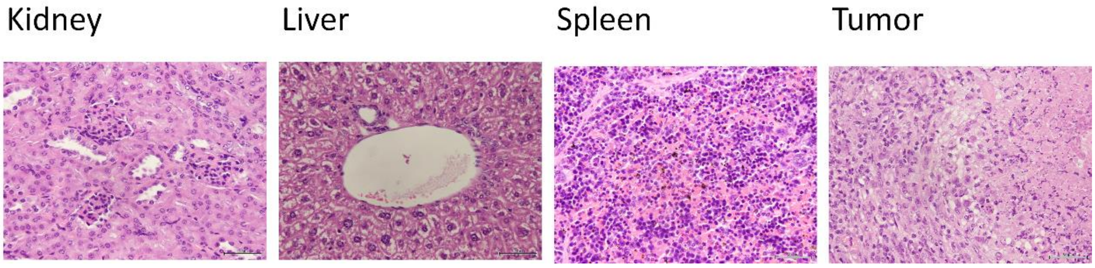

Figure S5. H&E staining of major organs after the magnetic hyperthermia treatment with FePt@IONP NPs.

Cell viability was assessed by using the MTT assay, which was based on the reduction of the dye MTT to formazan crystals, an insoluble intracellular blue product, by cellular dehydrogenases. MTT assays show that the mPEG-coated FePt@IONPs have no toxic reaction for KB cells, as shown in Figure S4. Furthermore, after magnetic hyperthermia treatment, we performed H&E staining, which reveals that normal cells are still live in the major organs but magnetic hyperthermia leads to apoptosis and necrosis in tumor tissue, shown in Figure S5. This result also confirms no cytotoxicity issue for FePt@IONP NPs.

S6: Stability of FePt@IONP NPs

(a) (b)

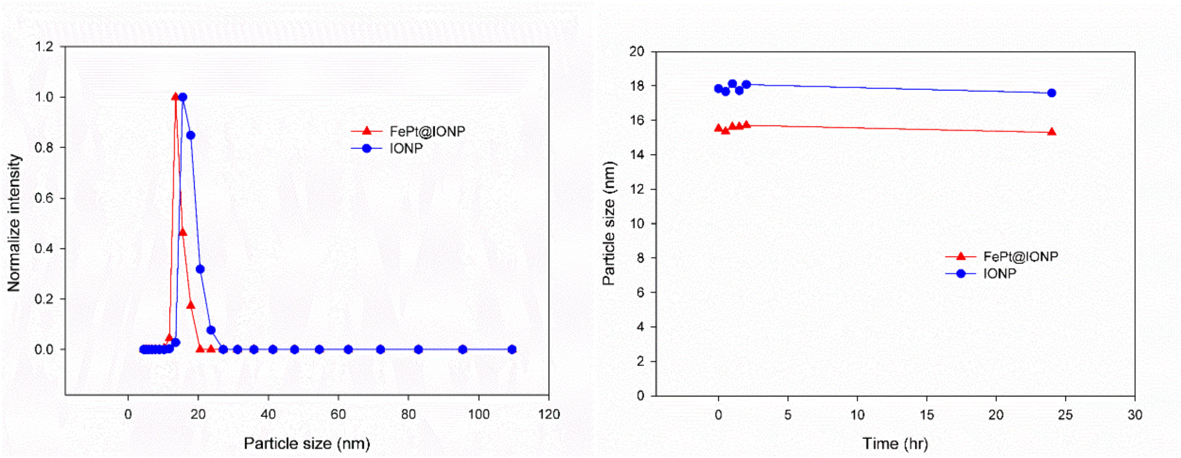

Figure S6 (a) Particle size distribution of FePt@IONP and IONP. (b) Variations of particle size with time.

Dynamic light scattering (DLS) is a well-known technique used for studying the stability of NPs. The DLS results reveal that the mean size of FePt@IONP is 15.5 nm and the size of NPs ranges from 11.7 to 20.5 nm (Figure S6a). The mean size and range of IONPs are slightly larger than those of FePt@IONP. No significant changes of mean size of both FePt@IONP and IONPs for 24 hrs, observed by DLS, indicating good stability of FePt@IONP and IONPs without aggregation (Figure S6b).

S7: Exchange-coupled properties of FePt@IONPs.

(a) (b)

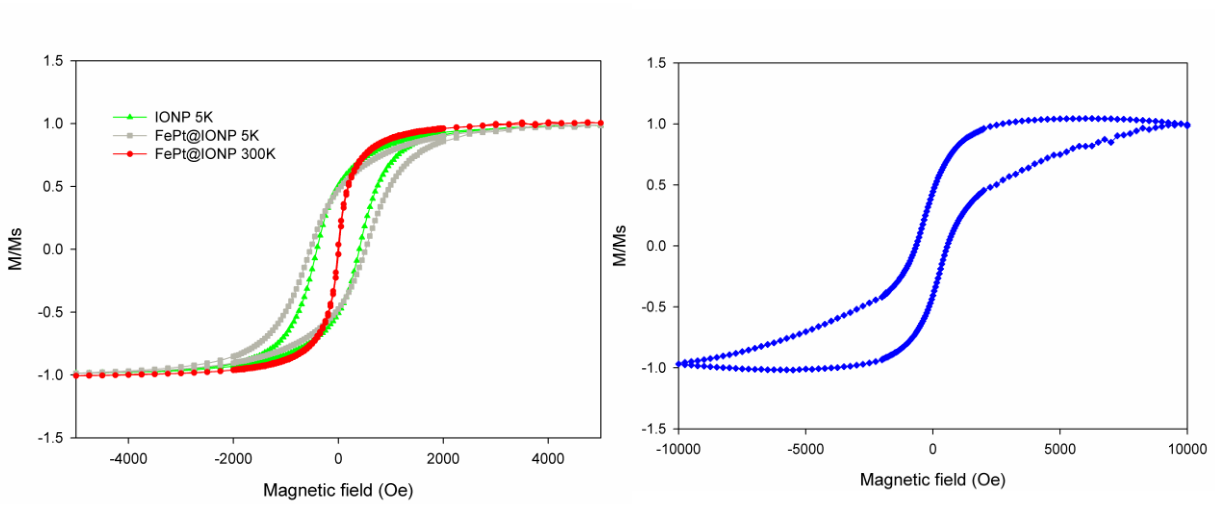

Figure S7. Exchange-coupled properties of core-shell FePt@IONPs were investigated by using M-H curve measurements with a superconducting quantum interference device (SQUID). (a) M-H curve of FePt@IONP at 300K and 5 K and M-H curve of IONP at 5 K . (b) M-H curve of FePt nanoparticles at 5 K. The red curve shows its superparamagnetic nature with zero coercivity at 300K. FePt@IONPs (grey curve) show larger coercevity than IONPs (green curve) at 5 K.

Since all nanoparticles are superparamagnetic at room temperature, to get information of exchange coupling between core and shell, we measure the hysteresis loops at 5K, shown in **Figure. S7a**. The disordered FePt core reveals a larger coercivity than IONPs and FePt@IONPs, shown in the inset of Figure S7b. Since the volume ratio of FePt core is only 1% of core-shell nanoparticles, the increased coercivity of FePt@IONPs compared to IONPs can be attributed to the presence of exchange coupling between soft Fe_3O_4 shell and relatively hard FePt core [4,5]

S 8: The fitting of the Imaginary susceptibility for IONPs and FePt@IONPs

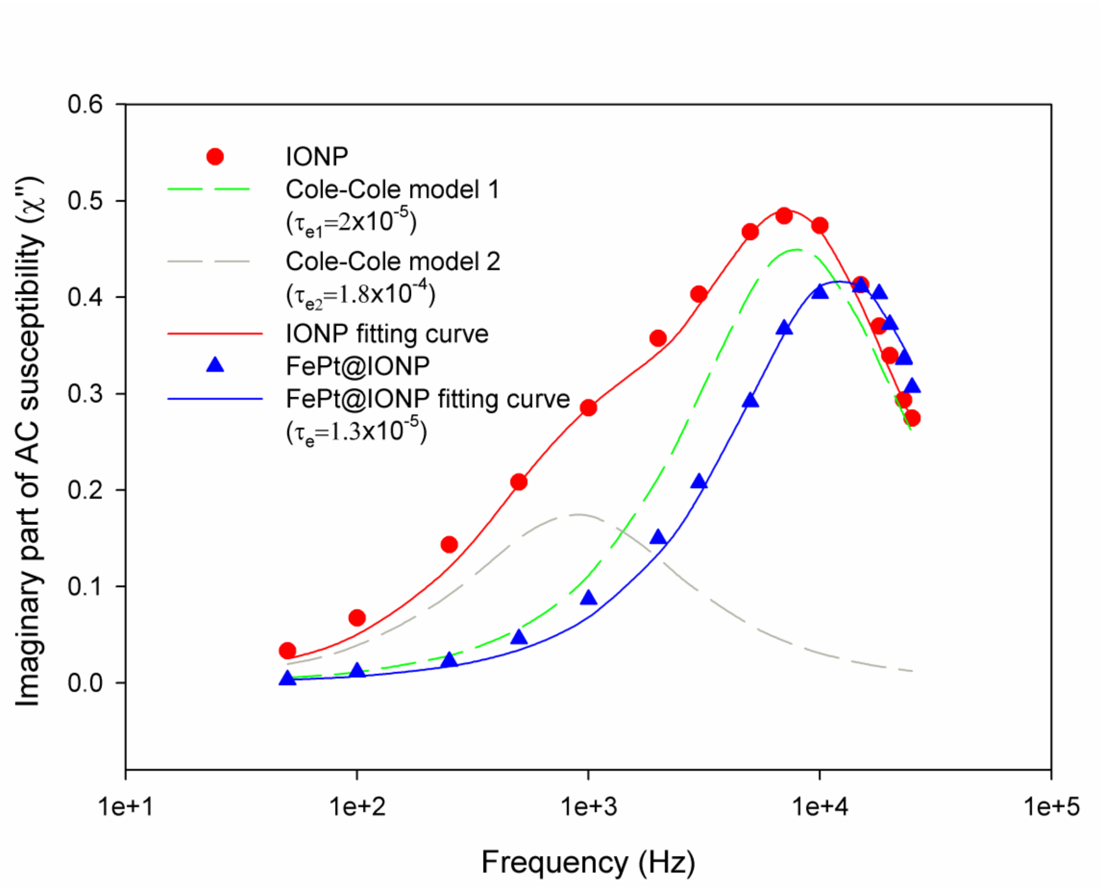

Figure S8. Imaginary susceptibility as function of frequency for IONP (red circle) and FePt@IONP (blue triangle). Using the Cole-Cole model with two symmetric peaks (gray and green dash line), we can fit the AC susceptibility curve of IONPs. Only one symmetric peak is needed to fit the AC susceptibility curve of FePt@IONP.

The information on interaction behavior can be obtained from AC susceptibility measurements $\chi''(\omega)$ by using empirical models. The symmetric $\chi''(\omega)$ can be conveniently represented by the Cole-Cole expression:[6,7].

$$\chi(\omega) = \chi'(\omega) + i\chi''(\omega) = \frac{x_0}{1+i\omega\tau_\epsilon} \qquad \text{(S2)}$$

where $\chi_0=NV^2M^2/K_BT$ is the static susceptibility of the sample comprising N monodisperse particles of volume V with a saturation magnetization M. The single relaxation time indicates the monodisperse particle. The AC susceptibility curve of FePt @IONP exhibits a symmetric

Debye peak, shown in **Figure S8**, indicating a suspension of monodisperse nanoparticles. The fitted relaxation time is $\tau_e = 1.3\times10^{-5}$ sec. On the other hand, the AC susceptibility of IONP exhibits a structure with features fitted by two relaxation times ($\tau_{e1}=2\times10^{-5}$ sec. and $\tau_{e2}=1.8\times10^{-4}$ sec.).

According to ref. [8], the effective relaxation time of $\sim10^{-5}$ sec corresponds to the particle size in the range of 15 to 20 nm. The larger particle or cluster size leads to the longer relaxation time. Our TEM images shown in Figure 2 of manuscript reveal that both of IONPS and FePt@IONPs have the particle sizes in this range and no obvious difference of size distribution for those two samples. Therefore, the longer relaxation time of IONPs ($\tau_{e2}=1.8\times10^{-4}$ sec) may suggest that a strong dipole interaction exists among IONPs, leading to lager magnetic clusters.

S 9: Micromagnetic simulations of the domain structure of (a) IONP and (b) FePt@IONP nanocube system

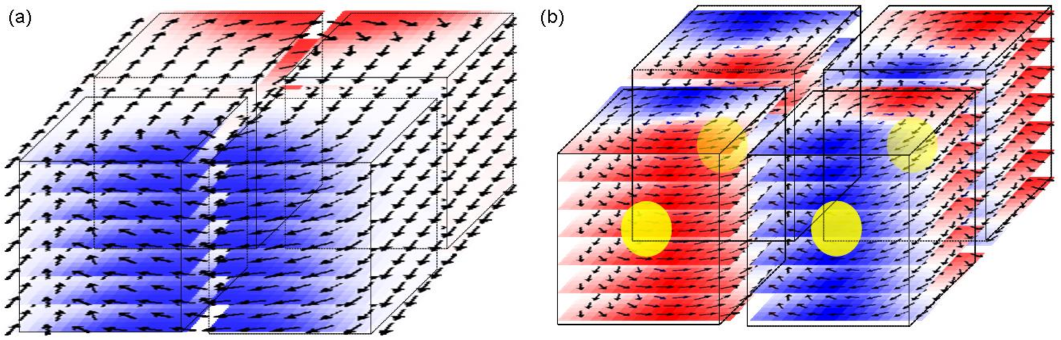

Figure S9. The OOMMF simulation for three-dimensional visualization of the magnetic moment within an assembly of four nanocubes. (a) Four Fe_3O_4 nanocubes form a flux closure state among nanocubes and (b) FePt@IONPs show that the presence of the core stabilizes the vortices in each cube.

The lateral sizes of the NPs were extracted from TEM images (the size of cubic IONPs is16.1nm; cubic FePt@Fe_3O_4 is 14.7nm with FePt core of 4nm). We use the micromagnetic OOMMF code to set the 3D magnetic configuration of the sample. The bulk saturation magnetization Ms (Fe_3O_4:4.71×10^5 A/m, FePt: 1.14×10^6 A/m), exchange constant (Fe_3O_4:0.3×10^{-12} J/m), cubic anisotropy constant (Fe_3O_4:0.1×10^5 J/m^3) and uniaxial anisotropy (FePt:7×10^6 J/m^3) were used for simulation parameters. The damping parameter of α= 0.5 was used to reach the equilibrium remanent state rapidly. The direction of the magnetic induction is indicated by black arrows. The OOMMF simulations for the remanent state reveal that the domain structure of core-shell FePt@IONP nanocubes exhibits a vortex state in each nanoparticle, analogously to the observed electron holography fringe patterns [9,10,11]. The induction state of IONPs shows strong interaction between adjacent nanocubes.

S 10: Shape evolution of magnetic nanoparticle assembly with or without a DC magnetic field

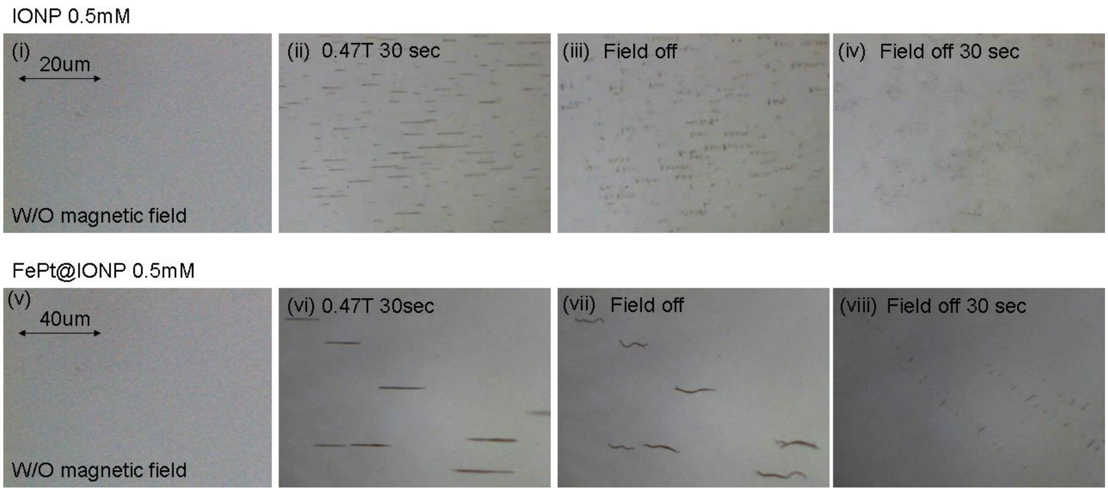

Figure S10. Shape evolution of magnetic nanoparticle assembly with or without DC magnetic field of 0.47 T by using optical microscopy.

Under the application of DC magnetic field, the magnetic particles can be assembled into different configurations [12, 13, 14, 15]. In our case, we set the DC magnetic field equal to 0.47 T. For IONPs: (i) well-dispersed nanoparticles without magnetic field; (ii) when the magnetic field of 0.47T was applied for 30 seconds, the chain clusters are formed and aligned along the magnetic field direction; (iii) when the magnetic field was turned off, the chain disappeared and a few clusters appeared in the aligned directions; (iv) at 30 seconds after turning off the magnetic field, the IONPs are re-dispersed. If water is slightly disturbed, IONPs can be well-dispersed in the solution, as shown in (i).

For FePt@IONPs: (v) the well-dispersed nanoparticles without magnetic field; (vi) when the magnetic field of 0.47T was applied for 30 seconds, the chain clusters are formed and aligned in the same direction as the field; (vii) when the magnetic field was turned off, the chain was still present but with distorted shapes; (viii) at 30 seconds after turning off the magnetic field, short-range chains remained. If water is slightly disturbed, FePt@IONPs can be well-dispersed in the solution, as in (v).

S 11: The AC susceptibility spectra for IONPs and FePt@IONPs in solution with or without a magnetic field

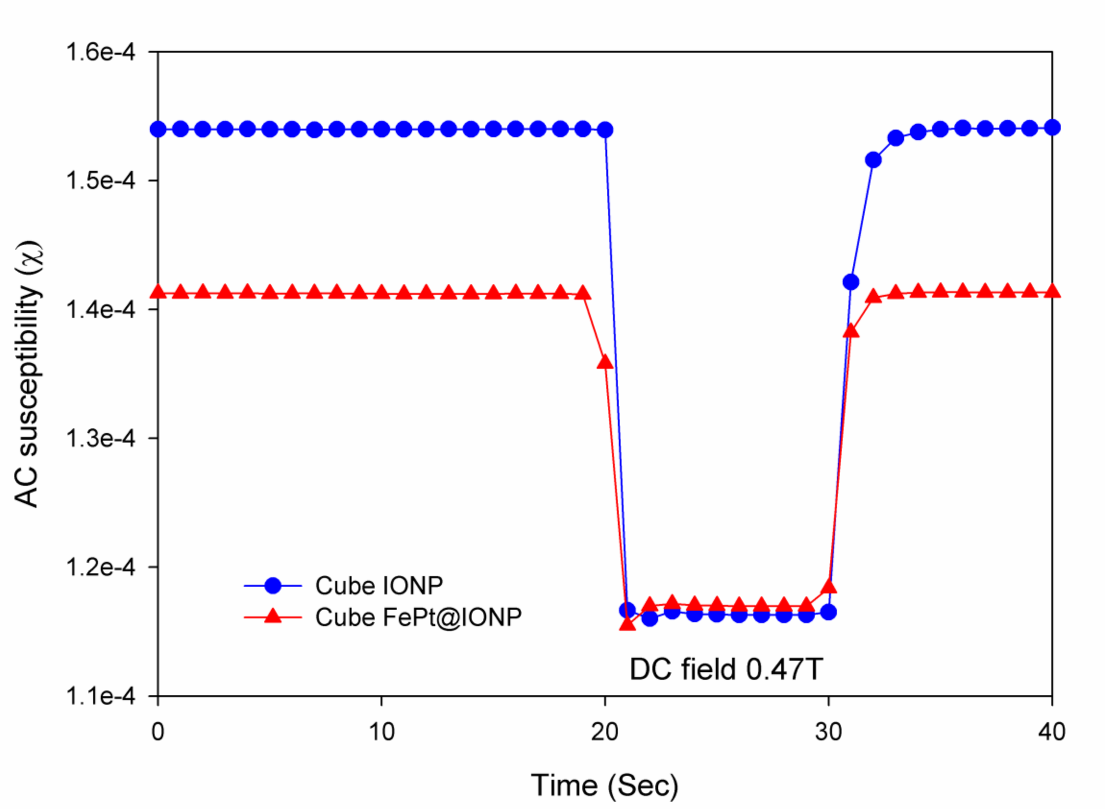

Figure S11. AC susceptibility spectra at 25 KHz of cube IONPs and FePt@IONPs with and without the DC magnetic field of 0.47T. The concentration of the sample is 0.1mg/ml. Here, the DC field is turned on and off at the 20th and 30th sec, respectively. With the applied filed, the nanoparticles aggregated, leading to reduced susceptibility. The reversible spectra indicate that both IONPs and FePt@IONPs can be re-dispersed after removing the DC field.

When the DC field is applied, the AC susceptibility of IONPs and FePt@IONPs in solution is reduced, as shown in **Figure S11**. When the field is removed, in both cases the AC susceptibility is raised to their original values. AC susceptibility can be reduced due to the formation of magnetic clusters [16]. When DC magnetic field was applied, aggregation of nanoparticles may occur, leading to a significant reduction in AC susceptibility. After removal of the DC field, the raised AC susceptibility indicates that the nanoparticles are well dispersed in the solution again.

S 12: Monte Carlo simulation of proton dephasing signal for continuous and discontinuous chains in large structures

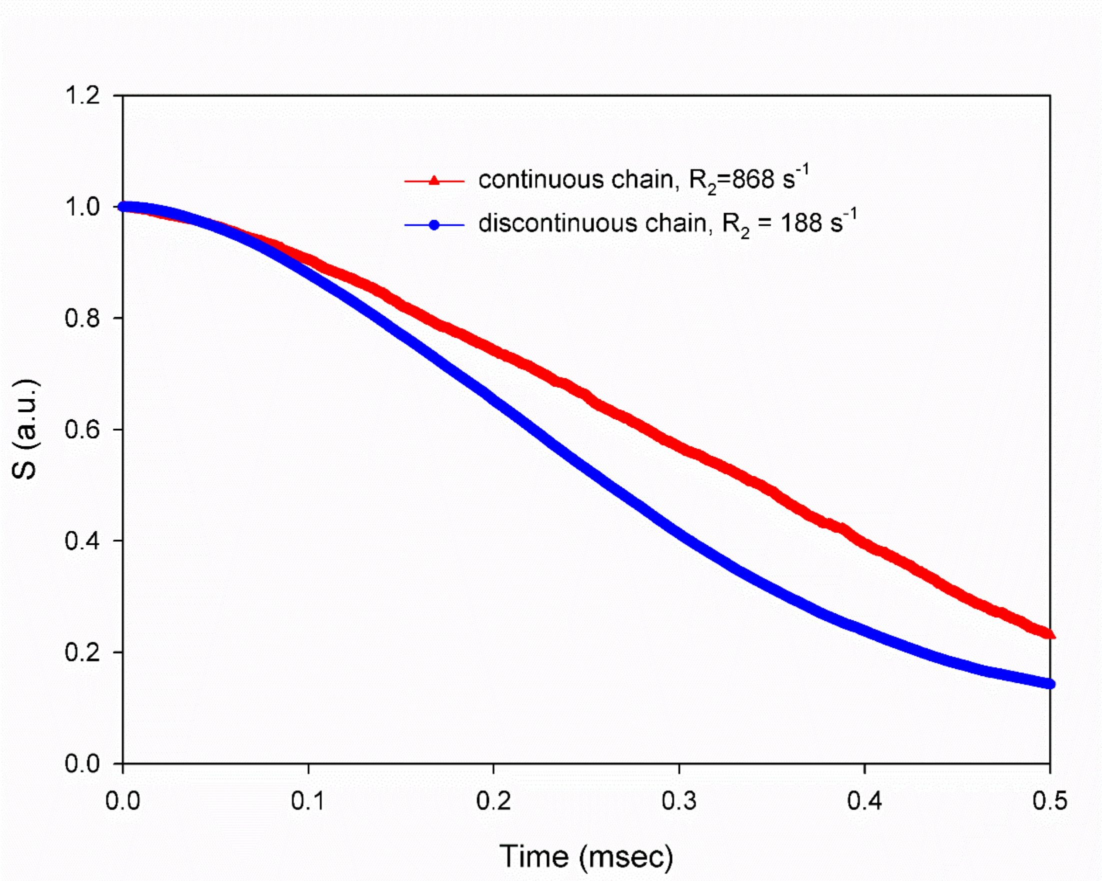

Figure S12. Proton dephasing signal S for two systems: continuous chains (blue curve) and discontinuous chains (red curve). The proton dephasing signal for large structures of continuous chain of 3636 magnetic nanoparticles (6x1x606 particle in xyz direction) and discontinuous chains of 101 magnetic clusters with each composed of 36 magnetic nanoparticles.

We consider a number of protons (Np) moving in a system with cubic Ns (Fe_3O_4 or FePt@Fe_3O_4). The dephasing of the proton moment S(t) is given by[17, 18]:

$$S(t+\Delta t) = \frac{1}{N_p}\sum_{i=1}^{N_p} cos(\varphi_i(t) + \Delta\varphi_i) \quad \text{(S3)}$$

$\varphi_i(t)$ is the angle with respect to the *z* direction.

$$\Delta\varphi_i = \gamma B(r)\Delta t \quad \text{(S4)}$$

γ is the gyromagnetic ratio of hydrogen (2.67×10^8 rad/sT). When calculating the net magnetic

field B due to the presence of nearby nanoparticles, only particles within distance of the proton are considered. The net magnetic field B can be expressed as:

$$B(r) = \frac{\mu_0 M}{3}\left(\frac{a}{r}\right)^3 (3\cos^2(\theta) - 1)) \quad \text{(S5)}$$

where μ_0 is the permeability of free space ($4\pi\times10^{-7}$ H/m), M is the magnetization of the particle$r$ is the distance from the particle center, θ is the angle with respect to the z direction and a is the size of the cubic nanoparticles. When the echo spacing is small, S(t) can be expressed by

$$S(t) = M \cdot exp\left(\frac{-t}{T_2}\right) \quad \text{(S6)}$$

where t is the overall time from the excitation to a particular echo we are measuring. S(t) has an exponential decay form with time.

Because of computational time, the simulation results shown in Figure 5 were done for small structures with a limited number of nanoparticles (total ~1500 nanoparticels). Our results illustrate the importance of the local configuration not just the global packing fraction. The relaxation rate depends also on the number of particle per cluster, on the local cluster density and the spacing between clusters. We performed also simulation for larger structures to ensure magnetic nanoparticle configuration more similar to experimental samples. The results are shown in **Figure S12**.

The configuration is set for the discontinuous and continuous chain structure, with each structure containing 3636 particles (total ~ 363600 nanoparticles). Due to large structures, the calculations are limited to a packing density of 0.001. From the curve fitting in the equation S6, the R_2 ($R_2=1/T_2$) of continuous chain is 868 s^{-1}, higher than discontinuous chain (188 s^{-1}) in the same packing density (Figure S12). Qualitatively the behavior is the same as for small clusters, in terms that the continuous chain has a larger R_2 than the discontinuous chain. Quantitatively, the values are smaller in comparison with the small structure system for

packing density of 0.001 (Figure 5). To compare the simulation results with the experimental data shown in Fig 1a, we extrapolate the R_2 value to 2mM Fe concentration (corresponding to packing density of 0.001) with the same linear relationship, the estimated R_2 values are 727 s^{-1}, 260 s^{-1} for FePt@IONPs continuous chains and IONPs discontinuous chains. On the other hand, the simulated values for the small structure are 1231s^{-1} and 415s^{-1}, respectively. For larger structures, the simulation results are closer to the real case.

S 13: Particle size enhanced relaxivity for FePt@IONPs

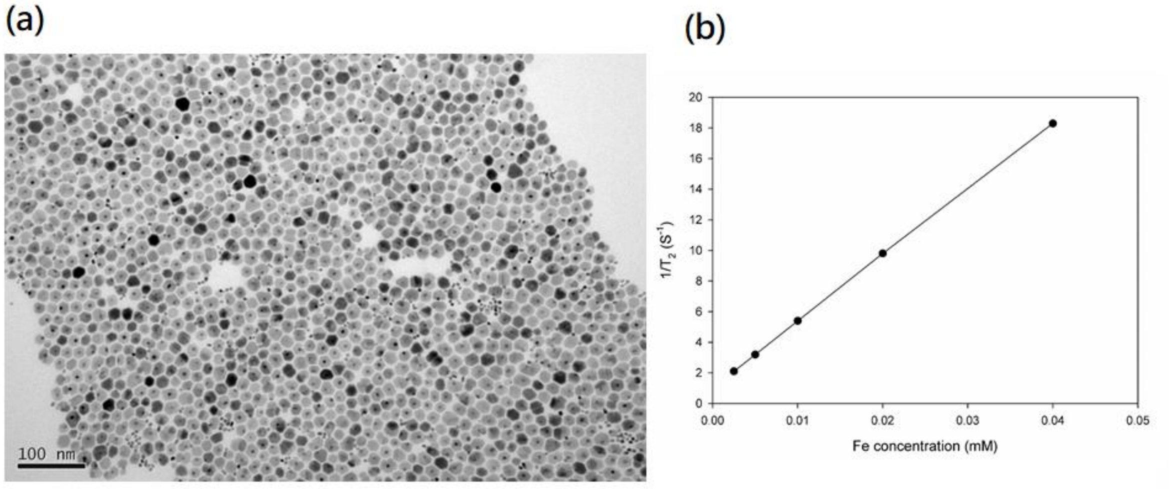

Figure S13. (a) TEM image of FePT@IONP and (b) relaxivity measurement with $1/T_2$ vs. Fe concentration.

By tuning the temperature of synthesis process, we can change the core-shell particle size. With increased size (17.8 nm) of superparamagnetic core-shell structure, we can further improve the relaxivity to 411.3 $mM^{-1}s^{-1}$, as shown in Figure S13. Although when the size is increased, the uniformity of NP becomes more difficult to control, our approach can still reach even higher r_2 by further optimization.

Supplementary Movie captions

Movie S1: The optical microscope movie was captured by charge-coupled device (CCD) cameras. Under the in-plane DC magnetic field of 0.47T, FePt@IONPs are aligned in the same direction as the DC field. The DC magnetic field is always applied during 5 seconds. The applied orthogonal AC pulse field (0.5 mT at 20 MHz) does not disturb the continuous chains of FePt@IONPs.

Movie S2: IONPs is aligned by the DC magnetic field of 0.47T. The applied orthogonal AC pulse field (0.5 mT at 20 MHz) disturbs the IONPs chains at regular intervals. The IONPs become discontinuous chains when AC pulse field is applied.

References

[1] J. P. Fortin, C. Wilhelm, J. Servais, C. Ménager, J. C. Bacri, F. Gazeau, *J. Am. Chem. Soc.* **2007**, 129, 2628.

[2] M. Suto, Y. Hirota, H. Mamiya, A. Fujita, R. Kasuya, K. Tohji, B. Jeyadevan, *J. Magn. Magn. Mater.* **2009**, 321, 1493.

[3] S. Dutz, R. Hergt, *Int. J. of Hyperthermia* **2013**, 29, 790.

[4] J. H. Lee, J.T. Jang, J. S. Choi, S. H. Moon, S. H. Noh, J. W. Kim, J. G. Kim, I. S. Kim, K. I. Park, J. W. Cheon, *Nat. Nanotechnol.* **2011**, 6. 418.

[5] B. G. Veronica, S. P. Regino, J. T. F. María, *J. Mater. Chem.* **2012**, 22, 2992.

[6] P.C. Fannin, B. K. P. Scaife, S. W. Charles, *J. Mag. Mag. Mat.* **1988**, 72, 95.

[7] K. S. Cole, R. H.Cole, *J. Chem. Phys.* **1941**, 9, 341.

[8] V. Singh, V. Banerjee, M. Sharma, *J. Phys. D: Appl. Phys.* **2009**, 42, 245006

[9] J. M. Thomas, E. T. Simpson, T. Kasama, R. E. Dunin-Borkowski, *Acc. Chem. Res.* **2008**, 41, 665.

[10] E. Snoeck, C. Gatel, L. M. Lacroix, T. Blon, S. Lachaize, J. Carrey, and M. Respaud, B. Chaudret, *Nano Lett.* **2008**, 8, 4293.

[11] L. M. Lacroix, S. Lachaize, F. Hue, C. Gatel, T. Blon, R. P. Tan, J. Carrey, B. Warot-Fonrose, B. Chaudret, *Nano Lett*. **2012**, 12, 3245.

[12] G. Singh, H. Chan, T. Udayabhaskararao, E. Gelman, D. Peddis, A. Baskin, G. Leitus, P. Král, R. Klajn, *Farad. Discuss.* **2015**, 181, 403.

[13] S. A. Corr, S. J. Byrne, R. Tekoriute, C. J. Meledandri, D. F. Brougham, M. Lynch, C. Kerskens, L. O'Dwyer, Y. K. Gun'ko, *J. Am. Chem. Soc.* **2008**, 130, 4214.

[14] S. Singamaneni, V. N. Bliznyuk, C. Binek, E. Y. Tsymbal, *J. Mater. Chem.* **2011**, 21, 16819.

[15] H. Jaganathan, D. L. Hugar, A. Ivanisevic, *ACS Appl. Mater. Interfaces* **2011**, 3, 1282.

[16] C.Y. Hong, C.C. Wu, Y.C. Chiu, S.Y. Yang, H. E. Horng, H. C. Yang, *Appl. Phys. Lett.* **2006**, 88, 212512.

[17] H. W. de Haan, *Magn. Reson. Med.* **2011**, 66, 1748.

[18] R. A. Brooks, F. Moiny, P. Gillis, *Magn. Reson. Med.* **2001**, 45, 1014.
